# Parental education level and ADHD diagnosis in childhood and adolescence: the moderating roles of gender, age, and family history of ADHD

**DOI:** 10.1007/s00787-025-02852-0

**Published:** 2025-09-18

**Authors:** Lotta Volotinen, Hanna Remes, Pekka Martikainen, Niina Metsä-Simola

**Affiliations:** 1 Helsinki Institute of Demography and Population Health, Faculty of Social Sciences, University of Helsinki, Helsinki, Finland; 2 Max Planck - University of Helsinki Center for Social Inequalities in Population Health, Helsinki, Finland; 3 Max Planck Institute for Demographic Research, Rostock, Germany

**Keywords:** ADHD, Age, Diagnosis, Family history, Gender, Parental education

## Abstract

Low parental education has been suggested as a risk factor for attention-deficit hyperactivity disorder (ADHD), but the associations may differ by gender and age, as boys are diagnosed more often and earlier than girls. Parental education might also not predict offspring ADHD diagnosis similarly if parents themselves have ADHD. We examined how maternal and paternal education level predicts offspring ADHD diagnosis at ages 4–17 and whether these associations are modified by gender, age (4–12 vs. 13–17), and family history of ADHD (biological parent and/or full sibling vs. none). We used data on 419,152 individuals born in Finland between 1994 and 2003, identified ADHD diagnosis from national registers with first clinical diagnosis or ADHD medication purchase, and estimated incidence rate ratios (IRR) in Poisson regression. Compared to tertiary education, basic parental education predicted a higher than twofold likelihood of ADHD diagnosis in offspring (maternal IRR 2.17, 95% confidence interval 2.07–2.28; paternal 2.36, 2.26–2.48). The likelihood was about threefold in childhood and twofold or less in adolescence, and mostly similar for boys and girls. Among those with family history of ADHD, the associations were weaker in childhood (highest IRR 1.85, 1.30–2.64) and negligible in adolescence. These findings suggest that low parental education is more strongly associated with an increased likelihood of offspring ADHD diagnosis in childhood than adolescence and in families without an identified history of ADHD. Improving diagnostic processes may help ensure appropriate access to diagnosis and care at any age, irrespective of parental education level and family history of ADHD.

**Supplementary Information:**

The online version contains supplementary material available at 10.1007/s00787-025-02852-0.

## Introduction

Attention-deficit hyperactivity disorder (ADHD) is a common neurodevelopmental disorder, estimated to affect about 5–7% of children and adolescents globally, although country-level variation in diagnosed cases is large [1–3]. ADHD diagnosis is defined by persistent challenges in attention regulation, hyperactivity, and impulsivity that were first observed in childhood [4]. These challenges may lead to a low educational attainment later in life [5, 6].

Previous studies have established that ADHD prevalence is elevated in childhood and adolescence for offspring of parents with low education [7–10]. Therefore, low parental socioeconomic position (SEP) has often been seen as a risk factor for offspring ADHD, but two recent studies that have used parental ADHD as a moderator have suggested that parental socioeconomic background may be less important in families with history of ADHD. A Danish register-based study found that the association between lower parental education and ADHD was weaker when parents had ADHD [8], and a small US survey-based study showed a similar moderating effect for parental income [11].

However, the measurement of parental ADHD is often incomplete, as many parents with ADHD have remained without a diagnosis due to development of diagnostic criteria in the recent decades. This undermeasurement of parental ADHD is a major limitation, as non-diagnosed parental ADHD may have led to a low education level in adulthood. Because ADHD is highly heritable [12], parents with ADHD symptoms are more likely than others to have offspring with ADHD, leading us to observe an association between low parental education and offspring ADHD diagnosis [13–15]. Thus, the identification of family history of ADHD in all first-degree relatives may allow for a more complete measurement of having ADHD in the family than the use of parental history alone.

Furthermore, both parental education level and family history of ADHD may together influence whether ADHD is diagnosed in childhood or adolescence. An earlier diagnosis may be likelier when ADHD is diagnosed in first-degree family members [16, 17], which may be in part explained by increased awareness of ADHD. In addition, higher parental SEP, which has been associated with elevated health literacy [18, 19], may also lead to earlier diagnosis through increased awareness. This increased awareness may be particularly important for girls, who are diagnosed less often and later than boys [20–23].

In the current study, we assessed how maternal and paternal education levels are associated with offspring ADHD diagnosis, how these associations are moderated by offspring gender and age, and further by family history of ADHD. We used high-quality Finnish register-based data on 419,152 individuals born in Finland during 1994–2003 and identified family history of ADHD of biological parents and full siblings.

## Methods

### Data and sample

Our data were based on individual-level observations of the Finnish total population from national registers. To construct our sample, we used datasets provided by Statistics Finland, which enabled the linkage of individuals with their biological parents and full siblings, with demographic information for the offspring and their parents. We identified ADHD diagnoses for the individuals and their family members with healthcare data from the Finnish Institute for Health and Welfare (THL), and medication purchases from the Social Insurance Institution of Finland (Kela).

Out of 588,295 individuals born in Finland during 1994–2003, we excluded offspring whose parents (9.8%) were born outside of Finland to avoid missing knowledge of ADHD medical history or parental education. For better comparability in terms of similar distribution of education and likelihood of ADHD diagnosis among the parental generation, we further excluded offspring with one or both parents who were born before 1960 or after 1979 (21.7%). Additionally, we excluded offspring whose both biological parents could not be identified (1.5%), leaving us with 423,271 individuals.

After these initial restrictions, we formed three samples: (1) all 4–17-year-olds, (2) 4–12 (childhood), and (3) 13–17 (adolescence). Those who were diagnosed with ADHD before age 4 were excluded from the first and second sample, and those diagnosed before age 13 from the third sample. Finally, we excluded individuals who died during the observed age interval (0.5% as 4–17-year-olds: 0.4% in childhood, 0.1% in adolescence) and individuals with missing information on any of the covariates at the beginning of the year of the individual’s 4th (0.7% for all 4–17-year-olds, 0.7% in childhood) or 13th birthday (0.9% in adolescence). In the final samples, 419,152 individuals were included in the follow-up at ages 4–17, 419,587 in childhood, and 408,390 in adolescence.

### ADHD diagnosis

We measured first ADHD diagnosis with a clinically recorded ADHD diagnosis or purchase of ADHD medication – whichever came first. A similar approach has been successfully used to measure ADHD in previous register-based studies [8]. Clinically recorded ADHD diagnosis (ICD-10: F90, ICD-9: 314) was measured from specialised inpatient (1969–), outpatient (1998–), and primary outpatient healthcare registers (2011–). ADHD medication purchases (Anatomical Therapeutic Chemical (ATC) classification system codes: guanfacine C02AC02; amphetamines N06BA01, N06BA02; methylphenidate N06BA04; atomoxetine N06BA09; lisdexamphetamine N06BA12) were identified from the National Prescription Register (1999–), which covers all retail pharmacies. Additionally, we measured the special reimbursement right of ADHD medication (national code: 331) from the Special Refund Entitlement Register of Medicines (2008–). Individuals with ADHD medication purchases who had a clinical diagnosis of narcolepsy or fatigue syndrome (ICD-10: G47.1, G47.4) were classified as having ADHD only if they also had clinically diagnosed ADHD.

### Parental education

We measured level of education separately for biological mothers and fathers from the population registers. We used the highest completed education or degree (International Standard Classification of Education (ISCED) categories: basic (ISCED 0–2), secondary (ISCED 3–4), and tertiary (ISCED 5+)), but supplemented tertiary education for parents who were currently studying for a tertiary degree. We measured maternal and paternal education at the beginning of the year of the offspring’s 4th (all 4–17-year-olds and in childhood) or 13th birthday (in adolescence) – hereafter referred to as baseline year.

### Offspring gender, age, and family history of ADHD

We measured offspring gender with sex assigned at birth (boys, girls) and formed the offspring age groups (4–12 vs. 13–17) using birth months and years. To identify family history of ADHD, we used all available information of biological parents’ and full siblings’ ADHD diagnoses and medication purchases until the individual’s 13th (in childhood) or 18th birthday (all 4–17-year-olds and in adolescence). Having a family history of ADHD was used as a binary variable (yes, no), if any first-degree family member had an ADHD diagnosis.

### Analysis

To estimate the association of parental education with offspring ADHD diagnosis, we used Poisson regression with robust error variance. This modified Poisson regression enables the direct assessment of relative risk when using a binomial outcome [24]. All models were adjusted for the baseline year (continuous) and parental birth cohort (1960–1964, 1965–1969, 1970–1974, 1975–1979) to control for changes in the diagnostic criteria, increases in ADHD diagnoses and medication use, and increase in parental education levels in Finland over time. Hospital district (*n* = 22) was added to control for area-level differences in diagnostic processes, ADHD medication prescriptions, and parental education. All models were run separately by maternal and paternal characteristics.

First, we estimated the associations of maternal and paternal education with offspring ADHD diagnosis amongst all 4–17-year-olds and tested whether the associations were moderated by gender, age, and family history of ADHD. Parental education’s interactions with gender, age, and family history were all statistically significant (*p* <.001) (see Online Resource 1, Table 1 for more information on interaction tests). Next, we stratified the models by offspring gender and age, and finally, we stratified the models further by family history of ADHD. The relative risks derived from the modified Poisson regression models are presented as incidence rate ratios (IRR) and 95% confidence intervals (95% CI). In addition, we present unadjusted incidence rates per 100 individuals in descriptive tables. We used Stata/MP 17 to construct the sample, conduct the analyses, and create the graphs.

### Additional analyses

In addition to the main analysis, we conducted five additional analyses. First, as lower household income has been associated with increased ADHD diagnosis in offspring [10, 11, 25], we adjusted for household disposable income (in annual quintiles) in the models for all 4–17-year-olds to assess whether the association between parental education and offspring ADHD diagnoses was independent of income. Second, we included parents that were not born in 1960–1979 to assess how this exclusion affected our results. Third, because the number and year of birth of siblings may affect the likelihood of identifying a family history of ADHD, with the lowest chances amongst singletons, we conducted a sensitivity analysis amongst two-child families in which both children were born in 1994–2003. Fourth, as changes in family structure and living arrangements may affect the association between parental socioeconomic position and offspring ADHD diagnosis, we focused further only on the intact two-child families. Fifth, to enable a comparison of our results with findings from earlier studies, we conducted a sensitivity analysis measuring only parental history of ADHD, as in the main analysis we identify family history of ADHD with both biological parents and full siblings.

## Results

In our sample, most parents had secondary education, whilst a quarter had tertiary education, and basic education was observed for only 11.6% of mothers and 16.0% of fathers. Amongst all 4–17-year-olds, the overall incidence rate of ADHD diagnosis was 4 per 100 individuals, more than 6 per 100 individuals amongst offspring whose mothers or fathers had a low education, and nearer to 3 per 100 individuals with the highest educated mothers or fathers (Table 1, see also Online Resource 1, Table 2 for the distribution of other covariates).

**Table 1:** Incidence rate of ADHD diagnosis (/100) and distributions (%) of maternal and paternal education and family history of ADHD

		Boys				Girls				All	
		4–12		13–17		4–12		13–17		4–17	
		(*N* = 214,596)		(*N* = 205,356)		(*N* = 204,991)		(*N* = 203,034)		(*N* = 419,152)	
		%	/100	%	/100	%	/100	%	/100	%	/100
	Total	100.0	4.2	100.0	1.8	100.0	0.8	100.0	1.3	100.0	4.0
Maternal	Basic	11.7	7.1	7.8	2.8	11.6	1.6	7.9	1.6	11.6	6.6
education	Secondary	63.7	4.1	60.3	1.7	64.0	0.8	60.5	1.2	63.8	3.8
	Tertiary	24.6	3.0	31.9	1.8	24.4	0.5	31.5	1.3	24.5	3.3
Paternal	Basic	15.8	6.8	13.6	2.8	16.2	1.4	14.2	1.7	16.0	6.2
education	Secondary	60.5	4.1	59.4	1.7	60.1	0.8	59.0	1.2	60.3	3.9
	Tertiary	23.7	2.5	27.0	1.5	23.8	0.5	26.7	1.2	23.7	2.9
Family history	No	97.2	3.7	95.5	1.6	97.2	0.7	95.0	1.0	94.9	3.3
of ADHD	Yes	2.8	20.2	4.5	7.1	2.8	5.5	5.0	6.2	5.2	16.3

**Fig. 1:**
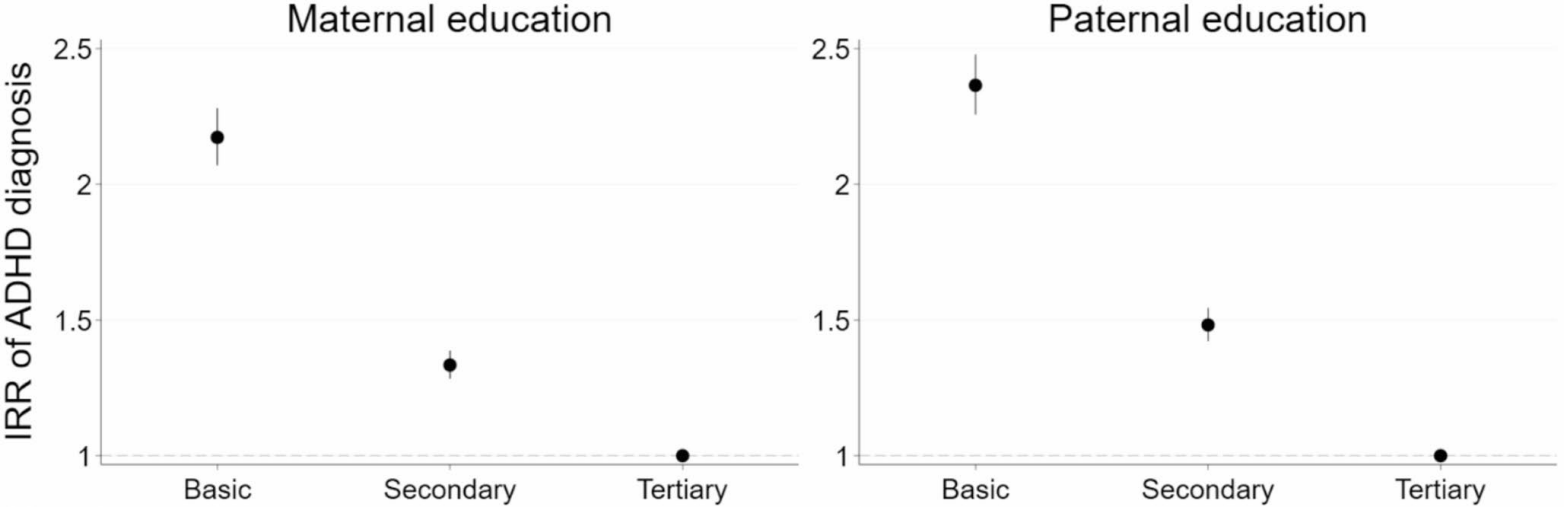
IRR of ADHD diagnosis for all 4–17-year-olds by maternal and paternal education (for model estimates see Online Resource 1, Table 3)

In the first model (Fig. 1), basic education of a parent was associated with a more than two-fold higher likelihood of an ADHD diagnosis for offspring in comparison to tertiary maternal (IRR 2.17, 95% CI 2.07–2.28) or paternal education (2.36, 2.26–2.48).

## The moderating roles of offspring gender and age

Boys received ADHD diagnoses more often than girls, with a gender ratio of almost 3:1. The timing of the diagnosis also differed by offspring gender, as diagnosis was much more often received before the age of 13 for boys, whilst girls were more often diagnosed in adolescence (Table 1).

**Fig. 2:**
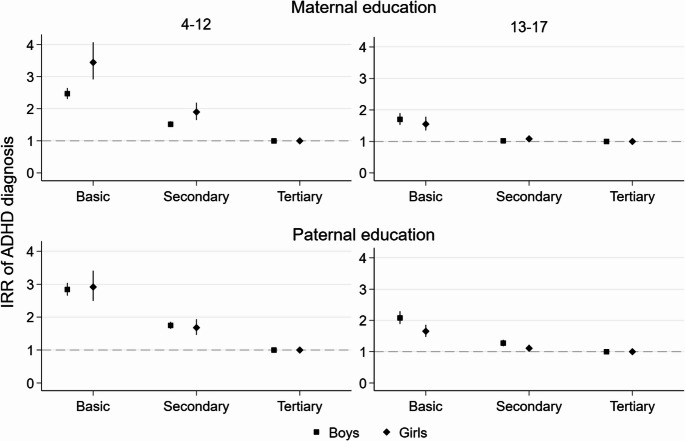
IRR of ADHD diagnosis by maternal and paternal education, offspring gender and age group (for model estimates see Online Resource 1, Table 3)

Lower parental education – both maternal and paternal – predicted higher incidence of offspring ADHD diagnosis amongst boys and girls in both age groups, but the associations were stronger in childhood than in adolescence (Fig. 2, see also Online Resource 1, Table 3 ). In childhood, when compared to offspring of tertiary-educated parents, girls whose mother had basic education had more than three-fold (3.46, 2.93–4.08) and boys more than two-fold higher probabilities (2.48, 2.32–2.65) to receive an ADHD diagnosis, whereas the probability was nearly three-fold higher amongst girls (2.89, 2.47–3.38) and boys (2.82, 2.64–3.02) whose father had basic education. In adolescence, boys whose father had basic education had about a two-fold higher probability (2.08, 1.88–2.29) of receiving a diagnosis as compared to boys with tertiary-educated fathers, whereas all other differences by parental education were smaller.

### The moderating role of family history of ADHD

Out of all individuals, 5.2% had a first-degree family member diagnosed with ADHD, and amongst them the incidence rate of ADHD diagnosis was about five-fold higher compared to individuals without a family history of ADHD (Table 1, see also Online Resource 1, Table 4 for the distribution of other covariates by family history of ADHD). The overall highest incidence rate of ADHD diagnosis was observed for boys with a family history (20.2 per 100 in childhood) (Online Resource 1, Table 5). Maternal and paternal education were on average somewhat lower among offspring with a family history of ADHD (Online Resource 1, Table 4).

**Fig. 3:**
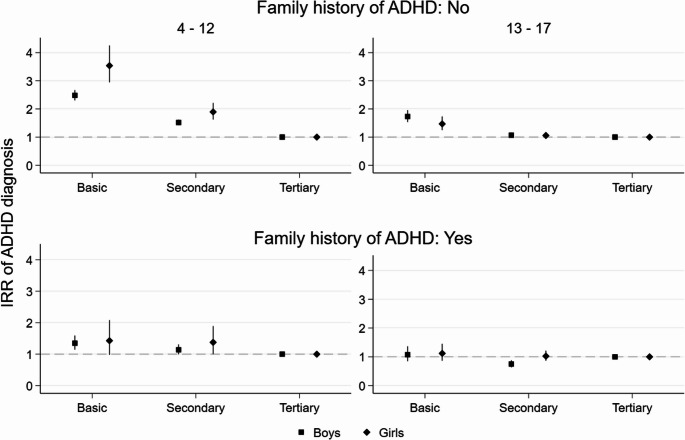
Maternal education and IRR of ADHD diagnosis by family history of ADHD, offspring gender and age group (for model estimates see Online Resource 1, Table 6)

**Fig. 4:**
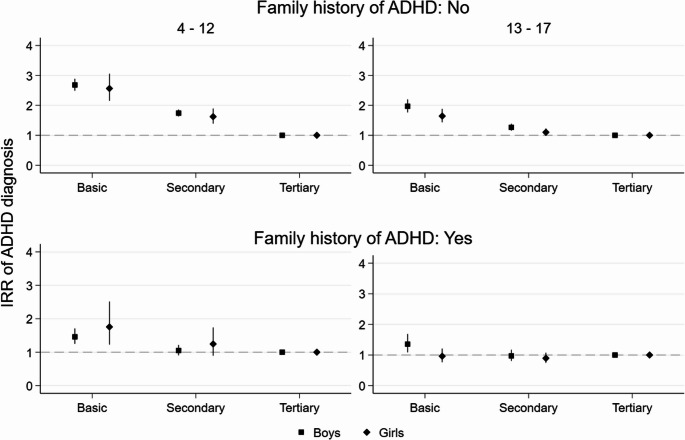
Paternal education and IRR of ADHD diagnosis by family history of ADHD, offspring gender and age group (for model estimates see Online Resource 1, Table 6)

In childhood, the association between lower parental education and offspring’s increased likelihood of an ADHD diagnosis was clearly weaker amongst those with a family history of ADHD than amongst those without it (Figs. 3 and 4). Differences in the probability of ADHD diagnosis by parental education were otherwise similar amongst boys and girls without a family history of ADHD, but in childhood, the differences by maternal level of education were larger amongst girls (IRR 3.54, 95% CI 2.94–4.25) than boys (2.48, 2.30–2.67). For boys and girls with a family history of ADHD, the associations of lower maternal and paternal education and increased probability of diagnosis in offspring were modest in childhood and negligible in adolescence. The only slight exception was that for boys with a family history, secondary maternal education was associated with a lower probability of ADHD diagnosis (0.75, 0.64–0.88) as compared to tertiary maternal education.

### Additional analyses

When household income was adjusted for in the models for all 4–17-year-olds, the associations between parental education and offspring ADHD diagnosis slightly attenuated but remained otherwise similar to the main results (Online Resource 1, Table 7). Removal of restrictions to the parental birth cohorts did not lead to any substantial changes in the results for all 4–17-year-olds (Online Resource 1, Table 8). When we tested the robustness of our results in two-child families, where both siblings were born in 1994–2003 (Online Resource 1, Table 9), or additionally restricted analysis to offspring living in intact two-parent families (Online Resource 1, Table 10), the associations of parental education and offspring ADHD diagnosis remained similar to the main results, although the differences somewhat attenuated amongst those without a family history. Finally, in the main analysis we used family history of ADHD – identified in both biological parents and full siblings, as the prevalence of parental ADHD is often underestimated due to lack of parental diagnoses. However, the use of parental history alone did not change our interpretation of the results (Online Resource 1, Table 11).

## Discussion

This study assessed whether the well-established association between parental education and offspring ADHD diagnosis is moderated by offspring gender, age, and family history of ADHD. Our results indicate that low maternal and paternal education are both associated with a higher likelihood of offspring ADHD diagnosis. Lower parental education predicts offspring ADHD diagnosis more strongly in childhood than in adolescence, and the associations are mostly similar for boys and girls. Furthermore, amongst boys and girls with a family history of ADHD, the association of parental education and offspring ADHD diagnosis was weaker – particularly in adolescence – in comparison to those without a family history.

As expected, the overall association of parental education and offspring ADHD diagnosis was similar to the findings from previous studies– lower parental education predicted increased diagnosis of ADHD in offspring [7–9]. In contrast to earlier studies that have used only a combined measure of parental education or solely maternal education [8–10], we used maternal and paternal education separately and noted that for both boys and girls, the probability of ADHD diagnosis increased quite similarly with both low maternal and paternal education. These associations were largely independent of household income, as they appeared only somewhat attenuated but otherwise similar when income was controlled for.

The association between lower parental education and increased probability of offspring ADHD diagnosis was moderate or even negligible amongst those with a family history of ADHD and stronger amongst those without it. These results corroborate findings from two prior studies that have measured family history of ADHD with parental history alone [8, 11], although it is often incomplete due to fairly recent development of diagnostics. A unique aspect of our study was the use of all diagnosed first-degree family members to identify a considerably larger share of individuals who had ADHD in the family, as the proportion of those with a family history of ADHD was 5.2% vs. 0.5% with parental history alone.

The difference between individuals with and without a family history of ADHD in the association of parental education and offspring ADHD diagnosis has multiple potential interpretations, such as (1) gene-environment interaction, (2) differences in the likelihood of receiving a diagnosis, (3) underdiagnosis of parental ADHD, or (4) selection bias. According to the first explanation, the observed differences might stem from gene-environment interaction, where low parental education increases the probability of offspring’s ADHD only among those with low genetic predisposition [11, 12]. Future studies should evaluate this mechanism by incorporating genetic data in their study designs. Second, it is possible that the findings reflect differences in the likelihood of receiving a diagnosis instead of differences in the probability of having ADHD. Although Finland has a universal healthcare system, the vast majority of children and adolescents attend public education, and schools offer regular health checks that should ensure equal access to ADHD diagnosis, it is plausible that parents may be differentially equipped to navigate the healthcare system [18, 19]. A family history of ADHD may bring about increased parental knowledge of ADHD in general, and awareness of having ADHD in the family, and these together with higher parental education level may improve parents’ ability to pursue a diagnosis when the offspring’s ADHD remains undetected by education and healthcare professionals. Hence, higher parental education could increase the likelihood of diagnosis particularly in families with a history of ADHD, narrowing the educational differences in ADHD diagnosis. This mechanism could be better assessed with more detailed data on ADHD symptoms and patterns of health care use, or with intervention studies. Third, given the high heritability of ADHD, the association of low parental education and higher probability of ADHD diagnosis amongst those without a family history of ADHD could also result from non-diagnosed parental ADHD, which has led to the parent’s lower adulthood educational attainment [5, 6, 15]. However, as the use of clinical diagnoses may yield an incomplete measurement of parental ADHD in register data, future studies could address this issue by screening parental ADHD instead. Finally, it is possible that underlying selective mechanisms are influencing the distribution of parental education amongst individuals with a family history of ADHD. For example, in our study, those with a family history had younger parents than those without a family history, and in general, educational attainment is on average higher in younger parental birth cohorts. To reduce this potential bias, we only included parents born in 1960–1979 in the main models. Nonetheless, the results remained similar in a sensitivity analysis in which we did not limit the included parental birth cohorts, indicating that this selection bias is unlikely to affect our results.

Furthermore, we are the first to show that amongst individuals with a family history of ADHD, the association between lower parental education and increased probability of receiving a first ADHD diagnosis is weaker in childhood and becomes almost negligible in adolescence, whilst amongst individuals without a family history the negative parental education gradient remains strong. This suggests that having knowledge of family history of ADHD may shape not only the probability but also the timing of offspring ADHD diagnosis. Having knowledge of ADHD in the family may facilitate a diagnosis in adolescence for those who were not diagnosed in childhood, and perhaps even more so among individuals with higher educated parents, which could explain the absence of the educational gradient. Still, it should be noted that the distributions of parental education in childhood and adolescence may be somewhat affected by different measurement times, as parents of diagnosed adolescents have had more time to achieve a higher education level.

Our results show also that boys are diagnosed more often and earlier than girls, which is in accordance with previous observations [20, 22, 23]. The gender difference may be explained with diverging development of ADHD traits, but also by gender bias in ADHD detection [19, 23, 26]. Girls with ADHD may have less difficulties in school, less externally visible and disruptive behaviour than boys, and hence, girls may be more likely to be diagnosed later or remain without a diagnosis [22, 23, 26, 27]. Although we had assumed that increased awareness of ADHD may be more important for girls than for boys, the associations between parental education and offspring diagnosis were mostly similar amongst boys and girls regardless of family history of ADHD. Nevertheless, more research is needed to investigate how parental characteristics might affect the timing of ADHD diagnosis.

### Strengths and limitations

A major strength of this study is the high quality of the Finnish register-based data used. We were able to form our sample from individual-level data that cover over 25 years, and to link the information of family members. Another strength in our study is the identification of all first-degree family members, which allowed us to identify families with ADHD, even when parents or earlier born siblings were non-diagnosed either because ADHD diagnosis did not exist or was less common in their childhood and adolescence.

However, whilst the data enable us to measure diagnosed ADHD over a long period, our measurement of ADHD lacks data on clinical diagnoses made in private healthcare, or from primary healthcare before 2011. Nonetheless, we have complete knowledge of ADHD medication purchases, and therefore, we are missing only those who never purchased medication. We estimate that the possible undercoverage for those born after 1994 is not large, as the overall incidence rate of ADHD diagnosis in our study is around 4%, which is close to the estimated global prevalence of 5–7% for children and adolescents and nationally estimated prevalences [28, 29]. For parents and older siblings who were born before 1994, the size of the undercoverage is less clear. Still, it should be noted that in Finland, ADHD diagnosis has become more prevalent only during the recent decades [28–30]. However, whilst the failure to identify all first-degree relatives with ADHD leads to some individuals being misclassified as not having a family history, the relatively low prevalence of ADHD means that they are only a small minority within the whole group without a family history, and thus, unlikely to cause any major bias. Moreover, the results remained similar when we used only parental history, considered only two-child families, and individuals living with both parents, suggesting that our findings are robust against model specifications.

To ensure a comprehensive and comparable measurement of offspring ADHD diagnosis, family history of ADHD, and parental characteristics, we chose to focus on individuals of non-immigrant background. In Finland, ADHD is more prevalent in families with immigrant background and given that these families are more often socioeconomically disadvantaged than others [31], our results likely marginally underestimate the association between parental education and offspring ADHD diagnosis. Additionally, as it is not possible to reliably differentiate between ADHD presentations (predominantly attention-deficit, predominantly hyperactive-impulsive, or combined) from our data, we were unable to assess whether the observed associations differ by the presence of hyperactive and impulsive behaviour.

## Conclusions

Our study adds to the previous evidence on parental education and offspring ADHD by observing family history of ADHD simultaneously with offspring gender and age. Our results suggest that parental education and family history of ADHD together predict the probability offspring ADHD diagnosis. Having a family member with ADHD diagnosis may increase the likelihood of ADHD diagnosis not just because of the hereditary elements of ADHD, but also due to factors that are related to the social aspects of receiving a diagnosis. Therefore, it is important that policies and diagnostic processes are developed to ensure that the access to diagnosis is equal for all, regardless of gender, age, parental resources, and knowledge of a family history of ADHD.

## Supplementary Information

Below is the link to the electronic supplementary material.

Online Resource 1 (XLSX 71.5 KB)

## Data Availability

The study uses data that are collected by register authorities (Statistics Finland, the Finnish Institute for Health and Welfare, and the Social Insurance Institution) and made available to researchers for specific research purposes stated in the research plan. All data used or produced by combining original data are confidential and the researchers cannot share them with third parties. Those interested can apply for a license to use the data for scientific research from the register authorities (Statistics Finland and Findata Health and Social Data Permit Authority).
